# Remarks on the Use of Electricity in Medicine and Surgery

**Published:** 1889-01

**Authors:** W. S. Tremaine

**Affiliations:** 217 Franklin Street; Buffalo


### *REMARKS ON THE USE OF ELECTRICITY IN MEDICINE AND SURGERY.*

By W. S. TREMAINE, M. D., Buffalo.

About ten years ago a prominent physician and teacher in New York said to me: "My experience with electricity has not been encouraging." Up to that time I had used electricity in an empirical way, as I have since learned it is generally used by the far greater number of medical men. The above remark, however, awakened inquiry, and since that time I have given the subject attention and not a little study and experiment. A somewhat extended observation has shown that physicians are apt to say, in cases that resist ordinary treatment, "try electricity," directing the patient to procure a faradic instrument and to use it. As well say to the patient "take opium," without any further direction, and expect beneficial results, if, indeed, the patient escaped disastrous ones.

But the prescription is usually a gratifying one to the patient, as the wonderful phenomena exhibited by electricity in its various forms are calculated to impress mankind with its occult powers. It is not then to be wondered at that the laity is greatly awed with its manifestations, and has clothed it with mysterious and extraordinary attributes in influencing all forms of vital phenomena, both vegetable and animal—indeed, with many of the *so-called intelligent* laity it is synonymous with *life* itself. Here, then, we have a favorable mental condition for the charlatan, and he is not slow to take advantage of it. I suppose my experience does not differ from that of the rest of my fellows, but I am occasionally called upon by some male or female, usually the latter, with a request that I will refer patients needing electrical treatment to her, as she has studied it under "Prof." So-and-So, or gone through a course at *such* an "institute" or "sanitarium." A very short cross-examination soon reveals the fact that such persons are about as well qualified to use electricity intelligently as a remedial agent, as to calculate an eclipse, or to take charge of the coast survey. No one but an exceptionally well-educated and experienced *physician* can intelligently *prescribe* electricity, and in many cases none other can *use* it to advantage.

It is no longer necessary to advocate, to such, its claims as a therapeutic and diagnostic agent.

In the hands of the general practitioner its application must be more limited, and, as I believe, for the following reasons: First, few physicians engaged in the arduous work of dealing daily with acute and grave maladies, can devote the necessary time to the theoretical and experimental study required to master electro-physics, electro-physiology, electro-diagnosis, and electro-therapeutics. Second, the appliances are numerous and expensive, requiring more or less care and attention. I think these two reasons coöperate often to discourage its employment in many cases where it would undoubtedly be of benefit. Opposed, as I am, to the multiplication of *specialties*, I have to admit that this one, viz., the skilful use of electricity in medicine and surgery, has its place; and to succeed, I believe that the *specialist* should have such mature judgment, diagnostic ability, and knowledge of the natural history of disease, as can only be obtained by experience in general practice, founded upon a good education.

When to this is added a thorough knowledge of electricity in its scientific aspects and practical application, he can scarcely fail to reach an extended field of usefulness, and sooner or later rescue electro-therapeutics from the opprobrium into which it has fallen in the estimation of many, on account of its misuse in the hands of charlatans and dishonest irregulars.

Not long ago one of the latter gravely assured me that he had perfected an arrangement by means of which he could send a current through a particular *strata* (*sic*) of the water in a bath-tub!

The founder of the science of electricity was a physician—Dr. Gilbert, of Colchester, Court Physician to Queen Elizabeth. In this connection it may be interesting to note that the rapidly-increasing modern appliances of electricity in the so-called useful arts, are but the practical application of scientific truths worked out in the laboratory and study by learned and trained minds, and are not, as many ignorantly suppose, new inventions accidentally stumbled upon, as a farmer's boy may evolve, during winter evenings, a patent clothes-pin, or a self-binding harvester.

The intelligent physician who aims at using electricity as a therapeutic agent, must familiarize himself with the nomenclature of modern electrical science, understand what is meant by electro-motive force, potential, current-strength, volt, ohm, ampère, farad, etc.; he will then be in a position to control and measure the agent he administers, and to select the apparatus required.

*Batteries.*—These are described in detail in all the various treatises on electricity, and differ not so much in principle as in detail for convenience of use. Their nature and form of construction must vary according to the particular work required of them.

The essentials of a good battery for the generation of the *galvanic* current for ordinary therapeutic purposes are, that it should possess sufficient electro-motive force, a constant and uniform current, last a long time, and require but little care; until recently these latter requirements have been imperfectly filled. The invention of the so-called "open-circuit" batteries has done away with much of the annoyance of these. The Leclanché cell or element may be taken as a type.

I have no desire to be invidious or to ally myself to any particular maker, but in my own work I have found the "Law" cell eminently satisfactory. Fifty or sixty cells of this form of battery will answer all requirements for office practice, including electrolysis. To this must be added a faradic induction coil, a rheostat, switch-board, or "current selector," an automatic rheotome, arranged for graduated slow and rapid interruptions, a pole changer, and last, but not least, a galvanometer or milliampèremeter, by which the dosage can be accurately measured. To these must be added the various forms of electrodes. Such an outfit will cost from $150 to $200.

*Storage or Secondary Batteries.*—Soon after the discovery of voltaic electricity it was found that the platinum or silver wires used to decompose saline solutions, acquired the power of giving a galvanic current of short duration. This led to the discovery of secondary batteries of feeble and imperfect form by Ritter in 1803. After much study and experiment on the part of various scientists, it was not until 1860 that a secondary element of power was constructed of lead by Planté.

The secondary or storage battery is now made of coiled lead plates set in a solution of sulphuric acid. The lead plates, by the action of the galvanic current supplied from a primary battery, such as Bunsen's or the gravity, become coated with the peroxide of lead, and acquire the property of storing a large quantity of electricity, which may be used for lighting or cautery purposes. When such effects are required, these so-called storage batteries furnish, perhaps, the most convenient form.

A standard of measurement for electro-motive force, internal resistance, etc., of batteries, as well as a test of accuracy of milliampèremeters is very much needed. I learn that Johns Hopkins University intends to establish a bureau for this purpose. It will be of great service to electricians.

*Electro-Therapeutics.*—It would be beyond the scope of this paper to enter into any extended discussion of electro-physiology and therapeutics. What is known on these subjects is extensively set forth in the various text-books and special treatises. The value of galvanic and faradic currents in the detection and treatment of the "reactions of degeneration" in the varied paralyses is now too well known to require any special advocacy. I merely remark that, as a rule, they can only be properly applied by a *physician*, and one who has given special attention to the subject and is supplied with the necessary apparatus. In some nervous diseases, where even the most sanguine admit that there can be no permanent curative action, amelioration of symptoms, and at least apparent improvement, may be obtained. This is sometimes the case in posterior spinal sclerosis (locomotor ataxia).

Two of such patients under my care, where the diagnosis was confirmed by Weir Mitchell, have been greatly benefitted by galvanic and faradic currents and the use of the faradic brush after the manner of Rumpf, of Dusseldorf.

The galvanic current sometimes produces striking effects in the relief of pain; in lumbago and sciatica it often acts like magic. It fails often from using too weak currents. The number of elements (cells) is no guide. Twenty or thirty of these will, in some batteries, show only a current strength of one or two milliampères, while, in my observation, cases upon which mild or faradic currents have no effect will yield immediately to twenty or thirty milliampères. In some neuroses of the stomach, electricity, in the form of the faradic current, proves beneficial. This will be more effective if one electrode is introduced into the stomach through the œsophagus. There is, possibly, some danger in this method, which my friend, Prof. Stockton, believes he has overcome by using a flexible tube, filled with a saline solution, as an electrode. He is now experimenting with a safe and convenient form of electrode for the stomach.

In various diseases of the pelvic organs in women, most marked beneficial results sometimes ensue, such as the removal of sub-involution and the plastic effusion resulting from pelvic cellulitis. In two cases of long standing, which had resisted other forms of treatment, I succeeded, recently, in causing absorption by a comparatively few applications of the galvanic current, ten to twenty milliampères.

That such plastic effusions are removed by the influence ot the galvanic current, is a fact. It is not quite easy to understand just how the current acts. It is attributed to what is called the catalytic or cataphoric action, which is akin to, or a mode of, electrolysis.

I have recently had under treatment a case of elephantiasis of the prepuce and skin covering the penis, which was thickened to five or six times its normal condition. The disease was of something over two years' duration. The scrotum was not involved. I made application of the galvanic current, one sponge electrode over the pubes, and the other (negative) at various points over the diseased structure, five to fifteen milliampères current strength, three times a week. At the present writing, about fifteen applications have been made. The disease has been removed, with the exception of some thickening of the prepuce, which has been materially reduced, and which I have every reason to believe will, under further applications of the current, entirely disappear. The disease in this form is rare, and the curative action of electricity most marked and decided. A report, more in detail, of this interesting case, I reserve for a future paper.

In displacements of the uterus, accompanied by sub-involution, both faradic and galvanic currents are indicated—the faradic current to exercise and tone up the muscles of the uterus (improperly called ligaments), and the trophic influence of the galvanic current for the engorgement and hyperplasia of the uterus.

But, perhaps, the most brilliant work lately done in electricity is that of Apostoli, in the use of strong currents applied directly to the diseased mucosa of the uterus in cases of endometritis, not only for its cautery effect, but, in addition, modifying favorably the nutrition of the organ.

I have seen some markedly favorable results from the use of the galvanic current in this way. In the treatment of uterine fibroids, the method of using electrolysis known as that of Apostoli, we have a decided addition to our means of dealing with these formidable growths. It is called by Englemann "the typical treatment for the reduction of neoplasms, especially of uterine fibroids, in which we utilize both the polar and the inter-polar effect."

The technique of the operation is explicitly set forth in recent periodical literature. It would serve no purpose to reproduce it here. The enormous current-strength required to accomplish the end, from 100 to 200 milliampères, should be sufficient to warn the inexperienced operator not to undertake it until he is thoroughly familiar with the force he has to use. A carelessness in allowing the electrode to come in contact with the speculum, produces a shock that no amount of persuasion will induce a patient to risk a second time.

There has been a great deal of discussion over the use of electrolysis in stricture of the urethra. I have no desire to be controversial, but I am quite satisfied, from my own observation, of its great value in this department of surgery. I *know* that some cases of stricture can be readily and safely cured in this way, without interference with the usual avocation of the patient. My earlier efforts in this direction were followed by disastrous results, and I was inclined to condemn; but after putting myself under the tuition of a medical gentleman who was successful in using this valuable method, I learned how to use it intelligently and with success. Why others have not been so fortunate, I do not feel called upon to explain.

In conclusion, we have in electricity a most powerful agent capable of modifying nutrition. As disease is a perversion of normal nutrition, it is a reasonable inference that, intelligently used, electricity is of great benefit in influencing disturbed vital action towards that normal condition which we call health.

In thinking of electrolysis and its catalytic effect, we are, perhaps, too apt to confine our attention to the *polar* action, or, in other words, to that chemical action which we can see and estimate in the immediate neighborhood of each electrode. We should bear in mind that electricity is *not* a current of fluid, but a force transmitted from the higher to the lower "potential," influencing the intervening particles of matter, be they organic or inorganic, living or dead. "Polarization," we call this in *inorganic* substances; why not polarization in living tissues; if that, indeed, may help us to understand the influence exerted. The subject is yet in its infancy, and worthy of the attention of the profession.

217 Franklin Street.

